# The challenges of marine spatial planning in the Arctic: Results from the ACCESS programme

**DOI:** 10.1007/s13280-017-0959-x

**Published:** 2017-10-26

**Authors:** Rosemary Edwards, Alan Evans

**Affiliations:** 1 Grove Down, Tutts Lane, West Wellow, Romsey, Hants SO51 6DY UK; 2 International and Strategic Partnerships Office, National Oceanography Centre, European Way, Southampton, SO14 3ZH UK

**Keywords:** GIS, Marine spatial planning, Ocean governance, Policy

## Abstract

Marine spatial planning is increasingly used to manage the demands on marine areas, both spatially and temporally, where several different users may compete for resources or space, to ensure that development is as sustainable as possible. Diminishing sea-ice coverage in the Arctic will allow for potential increases in economic exploitation, and failure to plan for cross-sectoral management could have negative economic and environmental results. During the ACCESS programme, a marine spatial planning tool was developed for the Arctic, enabling the integrated study of human activities related to hydrocarbon exploitation, shipping and fisheries, and the possible environmental impacts, within the context of the next 30 years of climate change. In addition to areas under national jurisdiction, the Arctic Ocean contains a large area of high seas. Resources and ecosystems extend across political boundaries. We use three examples to highlight the need for transboundary planning and governance to be developed at a regional level.

## Introduction

As sea-ice cover in the Arctic diminishes, the potential for future economic exploitation increases, most notably in shipping, oil and gas exploitation, fisheries and tourism. Failure to plan for cross-sectoral management could potentially lead to negative environmental impacts and user–user or user–environment disputes or conflicts (The Aspen Institute 2011). The Arctic Ocean is surrounded by five coastal states (Norway, Russia, USA, Canada and Denmark/Greenland) and contains a large area of high seas. Resources and ecosystems extend across political boundaries, highlighting the need for planning and governance to be developed and coordinated at a regional rather than national level.

Marine spatial planning (MSP) is increasingly used to manage the demands on marine areas, where several different users may compete for resources or space, and to ensure that activities at sea are as sustainable and efficient as possible. However, spatial planning for the future use of marine areas is a fairly new concept. Although marine areas are often regulated or allocated within individual economic sectors, there are at present few future looking and cross-sectoral examples of integrated marine spatial planning (Douvere 2008).

Marine spatial planning provides a practical way to organise the use of marine space and the interactions of its users, both spatially and temporally. Resulting marine spatial plans aim to balance the demands for development with the need to preserve the environment, while also achieving social and economic objectives. Many countries already designate or zone marine space, but conflicts can arise where management plans have been developed on a sector-by-sector basis, without sufficient consideration of the effects on other users or the environment. Successful MSP must take into account the spatial and temporal diversity of the sea, and understanding and mapping these distributions is a key step in the process (Crowder and Norse 2008). Marine spatial planning is a future-oriented process, offering a way to address and manage potential conflicts in advance, as well as predicting how these may change due to climate change or other pressures. Future accident/disaster scenarios can also be explored, planned for and mitigated as far as possible. Successful MSP can have significant economic, social and environmental benefits.

Although user–user conflicts in the use of maritime space may have significant adverse effects, some of the biggest concerns today are the impacts of human activities on the marine environment (user–environment conflicts). Several recent studies, including the Millennium Ecosystem Assessment (2005), have highlighted a continued decline in biodiversity in the world’s oceans. Cumulative impact of the effects of overfishing, pollution, habitat destruction and climate change are posing a significant threat to marine ecosystems and the delivery of ecosystem services (Worm et al. 2006; Crowder and Norse 2008; Halpern et al. 2008). Ecosystem-based management (EBM) is a governance and management approach which aims to maintain an ecosystem in a healthy, resilient and productive state (Stelzenmüller et al. 2013), which needs to also consider human uses. In 2011, the Arctic Council established an Expert Group on Arctic EBM, who produced a report “*Ecosystem*-*Based Management in the Arctic*” in May 2013. The report includes recommendation of a policy commitment, a set of principles for EBM in the Arctic and priority activities including the need to develop an overarching EBM goal for the Arctic Council.

An effective Marine Spatial Plan should apply EBM, balancing ecological, economic and social goals and objectives towards sustainable development. The plan should be integrated across all relevant sectors and agencies, both nationally and regionally, and should be adaptive and anticipatory, with focus on the long term, typically with a 10- to 20-year horizon. Marine spatial planning needs to be an iterative process that learns and adapts over time. The UNESCO’s Intergovernmental Oceanographic Commission (IOC) highlights six characteristics of effective marine spatial planning: (1) ecosystem-based, balancing ecological, economic and social goals and objectives towards sustainable development; (2) integrated, across sectors and agencies, and among levels of government; (3) place-based or area-based; (4) adaptive, capable of learning from experience; (5) strategic and anticipatory, focused on the long term; and (6) participatory, stakeholders actively involved in the process (Ehler and Douvere 2009).

International conventions are of importance for all maritime areas, including the Arctic. The 1982 United Nations Convention on the Law of the Sea (UNCLOS) is of relevance as it provides for the division of seas and oceans into maritime zones, some of which must be delimited by coastal states in order to have a legal effect. Equally of importance is the principle of freedom of navigation guaranteed under UNCLOS, which is conditional upon rules and standards on maritime safety and protection of the marine environment being met. Under UNCLOS Article 89, no state can unilaterally claim sovereignty or sovereign rights on the high seas and, as a result, cannot claim sole responsibility for MSP (Maes 2008). Although countries are committed to preventing harm to the environment and biodiversity beyond areas of national jurisdiction under UNCLOS and the Convention on Biological Diversity, few assessment procedures exist (Ardron et al. 2008). As a further complexity, the Arctic Ocean could also be classed as a semi-enclosed sea in accordance with the provisions of Article 122 of UNCLOS. In that case, under Article 123 of UNCLOS, the Arctic coastal states should seek to “coordinate the management, conservation, exploration and exploitation of the living resources of the sea” and “to coordinate the implementation of their rights and duties with respect to the protection and preservation of the marine environment” through an appropriate regional organisation.

The High Seas are a particular international component of the marine environment of the Arctic Ocean. Under the 1982 UNCLOS Convention, resources of the water column are available for exploitation by states external to the Arctic community (Part Vll), while sovereign rights to the exploitation of the resources of the underlying seabed and sub-seafloor may well belong to an Arctic coastal state (under Part Vl). This dual management regime is one which needs very careful planning and lends itself to the process of MSP.

## Pressures on the Arctic Ocean and the impacts of reduced ice cover

Arctic summer sea-ice extent has significantly reduced over the past 30 years (Serreze et al. 2007). Satellite measurements show earlier sea-ice break-up and later freeze-up, leading to greater periods of open water. Because of the extent of open water in September, ice cover the following spring is dominated by thin first-year ice, which will melt more readily than thicker multi-year ice (Stroeve et al. 2012). Milder winters and a decrease in the number of freezing days have also led to reduced ice thickness.

**Fig. 1:**
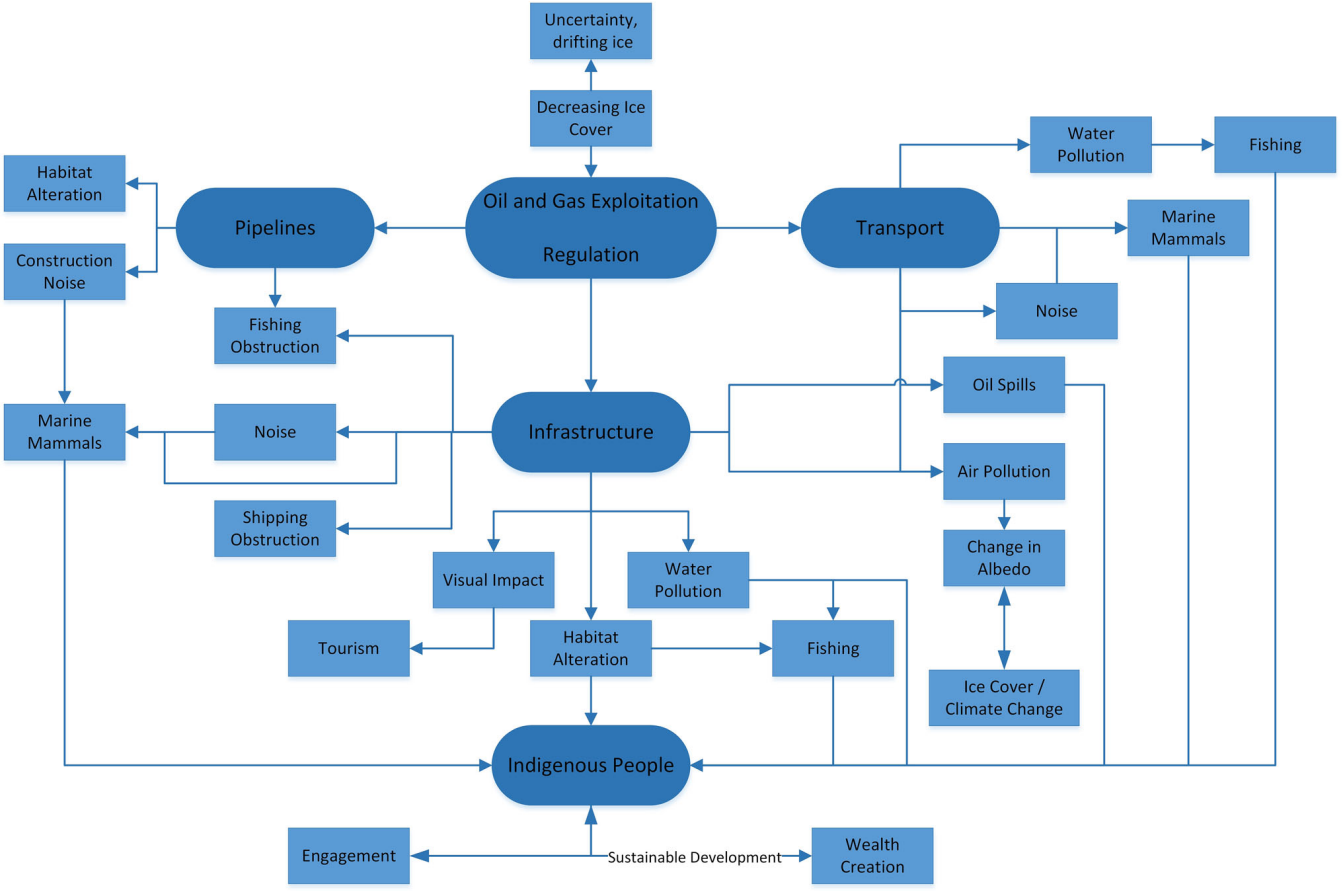
Example of some complex interactions/conflicts which may arise from increased hydrocarbon exploitation

Retreating summer sea ice is opening up new areas of the Arctic for potential economic exploitation. The U.S. Geological Survey carried out a hydrocarbon resource assessment of the Arctic and used models to conclude that about 30% of the world’s undiscovered gas and about 13% of the world’s undiscovered oil may be located north of the Arctic Circle (Gautier et al. 2009). Most reserves are likely under the continental shelf, with water depths of less than 500 m. However, successful submissions for continental shelf beyond 200 M, as enabled by UNCLOS Article 76 which provides the mechanism by which states can identify areas of sovereign rights beyond their 200 M Exclusive Economic Zones, will provide coastal states with access to resources further offshore. These changes, coupled with ever improving technology for gas and oil extraction in deeper water, could well lead to increased hydrocarbon exploitation in the Arctic. Interactions and potential conflicts between the hydrocarbon sector and other economic activities in the Arctic are likely to be complex and may result in negative environmental impacts (Fig. 1).

Exploitation of the Arctic’s hydrocarbon reserves would have implications both for the global climate and also for the Arctic environment. Oil spills, whether occurring as a well blow-out or during tanker movements, have potentially catastrophic consequences in the Arctic. The extreme weather conditions, remoteness and the corresponding lack of infrastructure present significant challenges. In addition, different mitigation and clean-up strategies will be required for open water or ice-covered areas. Many unknowns exist for the impact of a major oil spill on Arctic ecosystems and the people who rely on the Arctic Ocean for subsistence, and the fate of spilt oil in dynamic sea-ice conditions (The Pew Environment Group 2010). In May 2013, the eight member nations of the Arctic Council signed a binding agreement on Cooperation on Marine Oil Pollution Preparedness and Response. The agreement provides obligations and guidelines for cross-border collaboration in spill notification and response.

The decline in summer sea ice is also opening up the Arctic for shipping. The two main shipping routes, the Northern Sea Route and the Northwest Passage, are located largely along the shallow water continental shelf—areas of significant hydrocarbon prospectivity, and possible increasing conflict between different economic sectors. Increasing economic activity may have a significant detrimental effect on key cetacean species, e.g. bowhead whale *Balaena mysticetus*, fin whale *Balaenoptera physalus*, beluga *Delphinapterus leucas* and narwhals *Monodon monoceros*, through increases in underwater noise, pollution and danger of vessel strikes, while climate change may add additional stresses with changes in migratory patterns and prey distribution. This has important implications not only for conservation, but also for the local communities for whom marine mammals have both important resource and cultural significance. The number of cruise ships visiting Arctic waters has also increased significantly over recent years. The potential number of passengers aboard a cruise ship far exceeds the capacity of most search and rescue response vessels and aircraft in the Arctic. The Arctic Council’s Nuuk declaration of 2011 on Arctic Search and Rescue provides a binding agreement to coordinate the search and rescue coverage and response in the Arctic, but lack of sufficient onshore infrastructure (e.g. medical facilities, housing and food) to accommodate those rescued presents a significant challenge (Lloyds 2012).

Commercial fishing activities in areas beyond national jurisdiction in the central Arctic Ocean may also become significant as sea-ice cover decreases. The loss of ice cover will likely lead to increased primary production, zooplankton production and higher fish biomass throughout the Arctic region (MacNeil et al. 2010). Although some areas of the Arctic are covered by Regional Fisheries Management Organisations, current legislation is inadequate to fully protect areas which are now becoming accessible. Traditional fishing is another area where the effects of climate change coupled with increased economic exploitation may have negative impacts from multiple sectors.

Coastal states worldwide have started the process of MSP within waters under their jurisdiction, to integrate economic exploitation and social benefits with the duty to protect the marine environment and protect biodiversity. Examples of developing MSP initiatives in the Arctic include the Norwegian Barents Sea Integrated Management Plan and the Canadian Beaufort Sea plan. Integrated management is not so well developed for the remaining Arctic coastal states, with management and regulation operating on a sector-by-sector basis (Ehler 2014).

## Development of the ACCESS marine spatial planning tool

During the EU-funded ACCESS programme, an MSP tool has been developed, enabling the integrated study of information from all sectors under review (shipping, hydrocarbon exploitation, fisheries and tourism), and the associated human activities related to and within these sectors. It was beyond the scope of the ACCESS programme to produce a marine spatial plan, but instead to establish a framework with which interdisciplinary planning could be effected.

The MSP tool employs a Geographical information system (GIS), based on ArcGIS, to store, manage, interrogate and access the regulatory, spatial and temporal information outputs from the four theme areas. Two systems were developed, one desktop and a mirror online version. Users are able to visualise the various uses of marine space and identify overlapping activities. The MSP tool enables easy access to the regulation impacting on each sector which can be accessed as hyperlinks. Where future potential developments are envisaged, the MSP tool highlights the context and identifies the parameters of significance to the development allowing a prediction of any challenges.

**Fig. 2:**
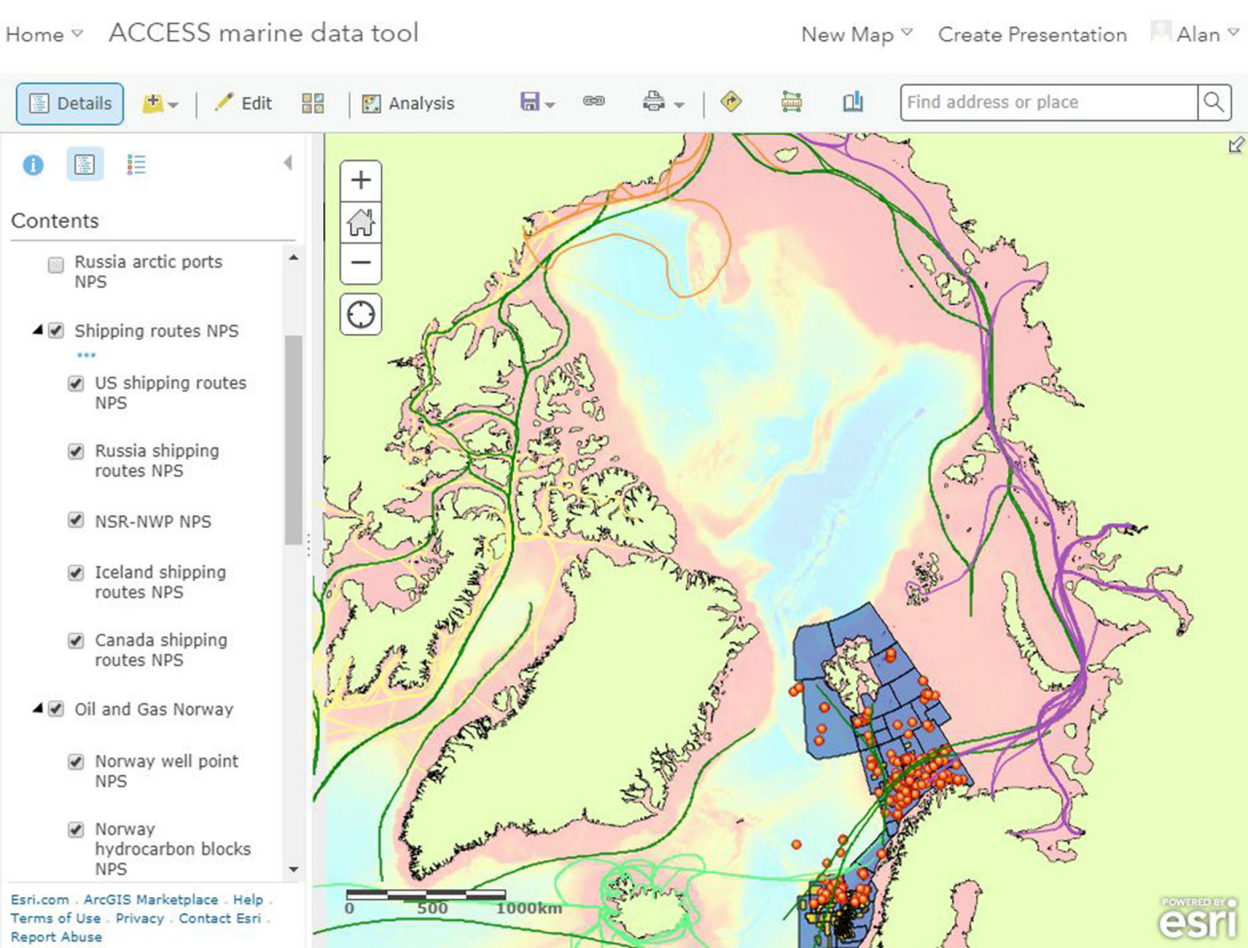
Example from the ACCESS ArcGIS online MSP tool

Figure 2 illustrates a screenshot of the online tool which reflects the types of information that are incorporated in the tool, and how these are organised within the GIS. Hyperlinks from shape files provide additional information relating to the selected layer. Visualising the spatial extent of sectoral activity and how these overlap provides a means of better managing those areas, and where environmentally sensitive areas are impacted by economic activity, the MSP tool can provide a means of planning the use of those areas. The MSP tool contains a combination of both relevant publically available data, as well as data and results generated by ACCESS partners.

**Fig. 3:**
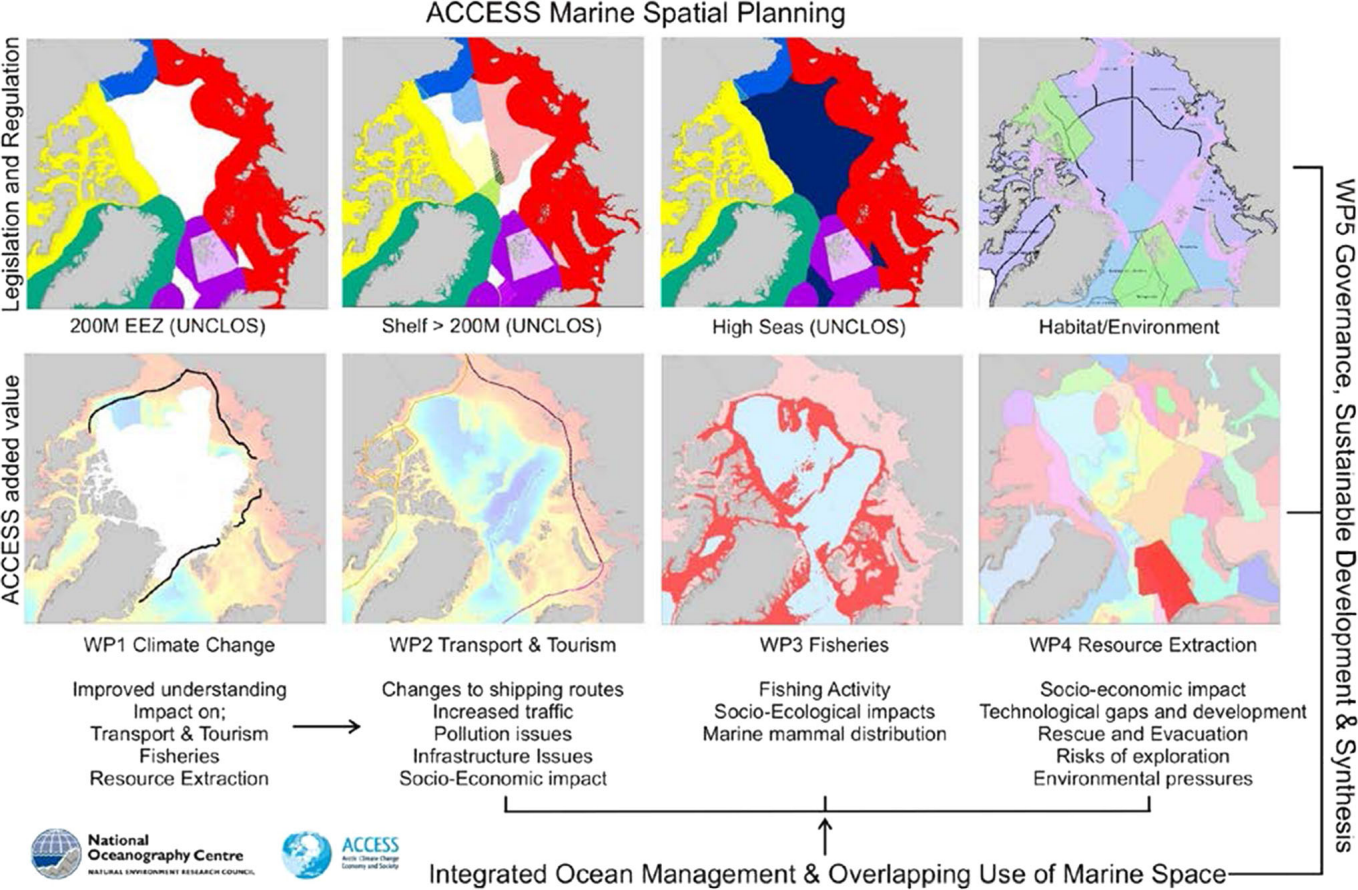
Schematic MSP tool functions within the framework of ACCESS (maritime zone areas indicative only)

To provide a schematic illustration of how the MSP tool functions within the framework of ACCESS, Fig. 3 shows eight plates representing different types of information. The four plates along the top illustrate the different maritime zones and regimes including the spatial extent of state sovereignty and of large marine ecosystems (LMEs). Legislative and regulatory documents are incorporated and can be accessed via the web and hyperlinks, and provide an accessible library of all relevant regulations for ACCESS partners. The four plates along the bottom represent the four work package theme areas within ACCESS. Each of these is populated with spatial data outputs from ACCESS and combined with regulatory information.

While the developed MSP tool covers the whole Arctic Ocean region, it is not possible to deliver detailed analysis for the entire region (largely due to lack of available data for some areas). Selected areas and themes have been targeted to demonstrate the MSP tool. The first example sets the context over the Arctic Ocean, while the following two examples look at regions under pressure from increasing economic exploitation: the Barents Sea, and the Chukchi Sea and Bering Strait region.

## The Arctic Ocean: regional context

The effects of climate change are apparent throughout the Arctic, with rising temperatures leading to a decline in sea-ice volume and changes to weather patterns, season length and ecosystems. The Arctic Ocean contains a range of different jurisdictional areas, including high seas and the Exclusive Economic Zones of the five Arctic coastal states. Current legislation ranges from supranational conventions, regional multinational or bilateral agreements, and finally national legislation. In addition to binding legislation, different sectors of the Arctic are also covered by various non-binding guidelines, codes and resolutions, for example the Arctic Offshore Oil and Gas Guidelines 2009 produced by the Arctic Council. This complex hierarchy and geographic coverage has led to jurisdictional and sectoral fragmentation of governance covering the Arctic Ocean (Young 2016). MSP allows the mapping and analysis of these different governance regimes, as well as living and non-living resources, species distributions and habitats, all of which may extend across political and jurisdictional boundaries.

**Fig. 4:**
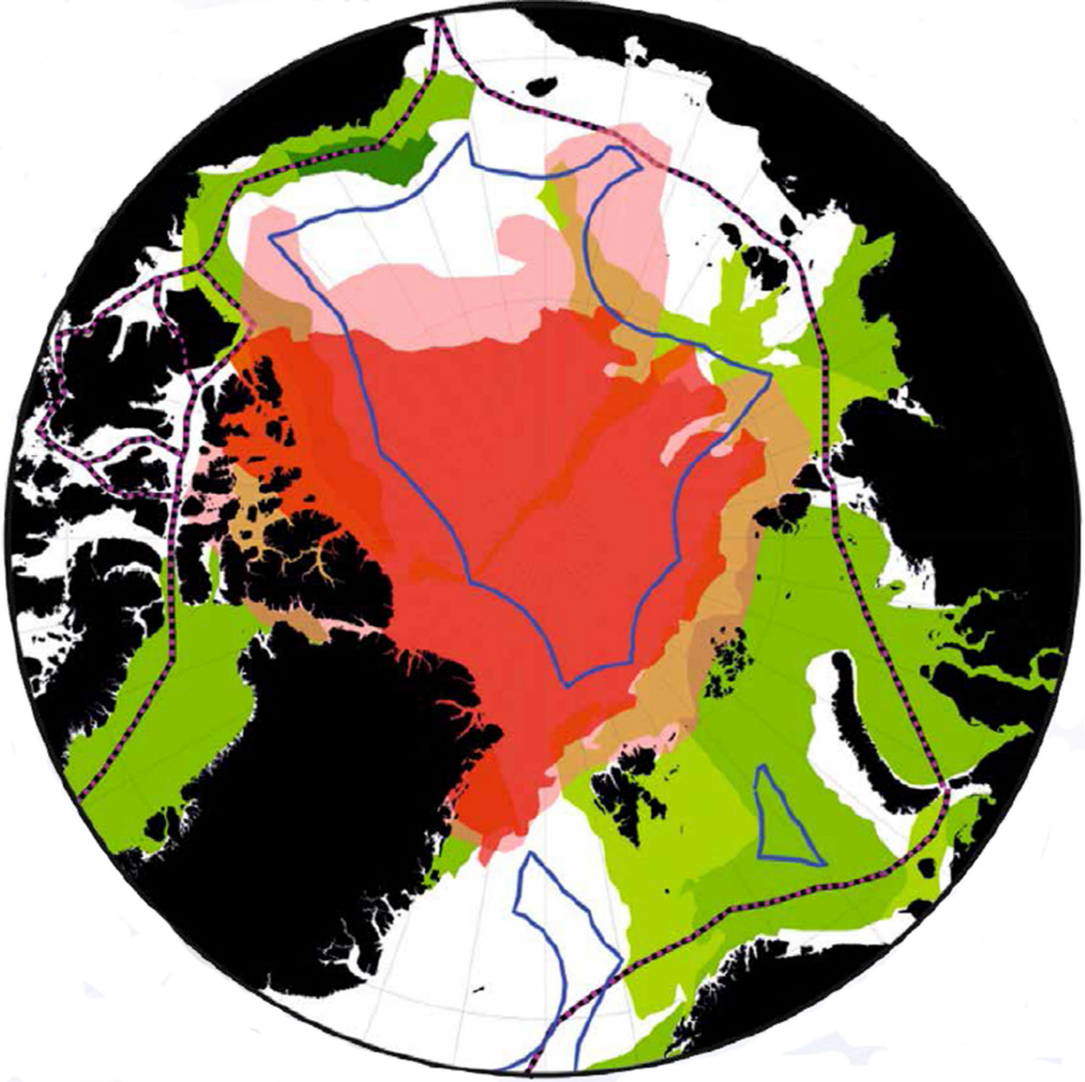
The Arctic Ocean showing areas of potential conflict. Pink shaded area shows the sea-ice extent in September 2010, while red shows the decreased extent in September 2012. Geological provinces in the Arctic with estimated significant undiscovered oil are shown in green (light green shows low potential, to dark green representing the highest potential). Blue line shows coastal states 200 M limits, while pink and black dashed lines show the Northern Sea Route and North West Passage, respectively

Retreating summer sea ice is allowing access to more areas of the Arctic Ocean both within and beyond national jurisdiction. Future economic development of these areas from a range of users may lead to possible cross-sectoral conflicts over the region as a whole (Fig. 4). Increased hydrocarbon exploitation is recognised as a significant possible future challenge to balancing socioeconomic effects and environmental protection in the Arctic (The Pew Environment Group 2010). Decreased ice cover as well as improved technology would allow oil and gas extraction in ever deeper water. Although a number of non-binding guidelines have been developed, for example by the Arctic Council, there is no regional legislation for hydrocarbon exploitation, and regulation will be covered by the individual state within whose EEZ the development takes place. However, oil spills present the largest threat to the Arctic marine environment and have the potential to spread over many hundreds of kilometres and jurisdictional boundaries. The Arctic Council agreement on Cooperation on Marine Oil Pollution Preparedness and Response (EPPR), signed by the eight member states in 2013, provides operational guidelines, although these are non-binding.

In addition to hydrocarbon exploitation, retreating sea ice is also opening up the Arctic to shipping. The two main shipping routes the Northern Sea Route and the North West Passage are both located largely along the shallow water continental shelf, where the USGS have identified significant hydrocarbon prospectivity (Gautier et al. 2009)(Fig. 4). Clearly, the potential exists for user–user conflicts in these areas. User–environment conflicts with a negative impact on biodiversity may also arise due to the potential introduction of invasive species via ballast water or hull fouling.

While many of the predicted increases in economic activity will take place over the continental shelf or in coastal state’s EEZs, as the central Arctic Ocean becomes ice free, coupled with possible northward migration of fish stocks, this allows the potential for commercial fishing activities in the areas beyond national jurisdiction. Although some parts of the Arctic Ocean are covered by a Regional Fisheries Management Organisation (RFMO), for example the North East Atlantic Fisheries Commission (NEAFC) involving Denmark/Greenland, Norway and Russia as Contracting Partners which covers the Arctic from 42°W to 51°E longitude including a small area of High Seas in the central Arctic, many areas are not covered by any regional legislation. Current legislation is inadequate to fully protect these areas. Options to regulate potential emerging fisheries include the establishment of a new RFMO or arrangement for the Central Arctic Ocean (see, for example, Molenaar 2014).

Successful establishment of continental shelf areas beyond 200 M under UNCLOS Article 76 will also provide coastal states with sovereign rights to sub-seabed resource exploitation. This presents a further complication in that any coastal state successful in securing its exploitation rights on the seabed and within the subsoil would be in potential conflict with those states seeking to exploit the resources in the superjacent water column under the regime of the high seas. The operation of this dual legal regime has largely been untested, but any instances will have great significance for the Arctic Ocean in the decades to come.

## The Chukchi Sea and Bering Strait

**Fig. 5:**
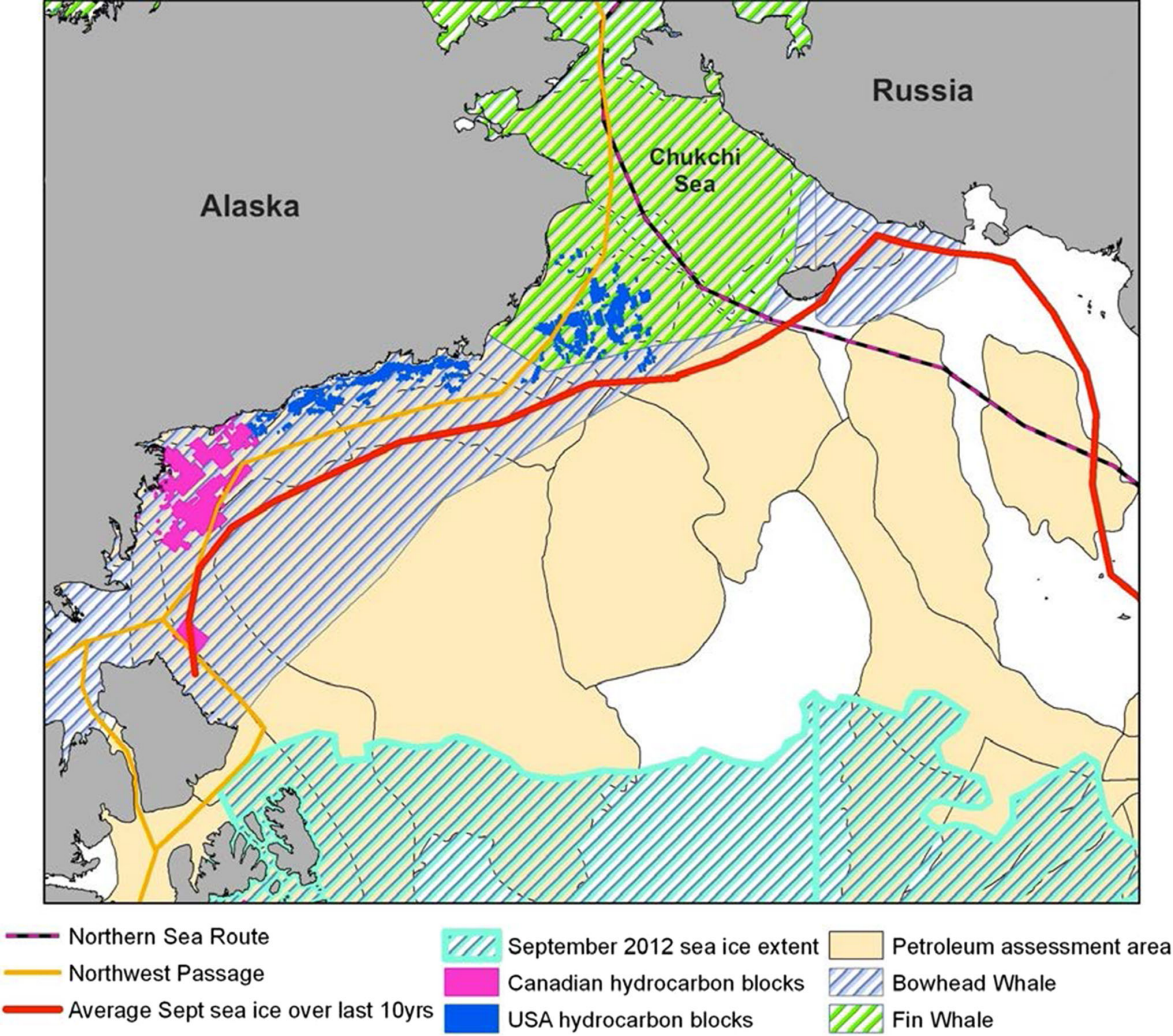
The Bering Strait and Chukchi Sea region. Blue and green hatched areas show bowhead and fin whale ranges (IUCN Red List), hydrocarbon blocks are shown in blue and pink, while the orange and pink/black lines show the North West Passage and Northern Sea Route shipping routes, respectively. Tan coloured areas show potential hydrocarbon provinces of the USGS Circum-Arctic Resource Assessment (Gautier et al. 2009). The September 2012 sea-ice extent is shown by the hatched area outlined in pale blue

The second case study looks at the Chukchi Sea and Bering Strait region (Fig. 5). From south to north, the Bering Sea, Bering Strait and Chukchi Sea provide the linkage from the North Pacific to the Arctic Ocean. At its narrowest point, the Bering Strait is only 80 km wide and represents a “pinch-point” between the Pacific and Arctic Oceans. Hydrocarbon exploration and exploitation in the United States and Canadian Chukchi and Beaufort Seas is increasing, while in the Russian East Siberian Sea Rosneft and ExxonMobil have agreed joint licence areas. Figure 5 outlines some of the user–user and user–environment conflicts that may result from increased hydrocarbon exploitation. In addition to environmental risks associated with the oil platforms and drilling/extraction activities (e.g. oil spills, acoustic noise, pollution), increased shipping activity (supply vessels, rig movements) through the Bering Strait is inevitable.

Increased commercial transit shipping traffic along the Northern Sea Route and North West Passage must all either exit or enter the Arctic through the Bering Strait, and hence within the ranges of the bowhead and fin whale in this area of the Arctic (Fig. 5). The bowhead whale, along with belugas and narwhals, are present in the Arctic all year round and are significantly affected by changes in their environment caused by climate change (Reeves et al. 2013). According to Reeves et al. (2013), more than half of the Arctic range of these three whale species overlaps known or suspected offshore hydrocarbon provinces. Hydrocarbon exploration and exploitation leads to significant increases in underwater noise, while increasing vessel traffic escalates the risk of ship strikes, pollution and noise.

Increased economic activity in these areas, coupled with changing climatic conditions (which could lead to changes in migratory patterns and prey distribution), has therefore significant implications not only for the conservation of the cetacean species and their habitats, but also for the local communities that depend on marine mammals for both food supply and cultural cohesion. Through careful planning of shipping lanes, and temporal or spatial closures of feeding or calving areas (for example to oil and gas drilling), MSP could prove a vital tool to mitigate against the impacts of human activities on Arctic cetaceans, for example the reduction in sources of underwater noise.

## The Barents Sea

The Barents Sea is an area of rich living natural resources, while also experiencing growing exploitation of hydrocarbon resources, and an increase in maritime transport. As a result, this is an area where coordination and regulation of these activities is required to manage interactions between different economic sectors, and also with the natural environment. An integrated management plan is already in place for the Norwegian Barents Sea–Lofoten area, integrating fisheries management measures with those for oil and gas, transport and nature conservation. The plan covers Norwegian waters only; no marine spatial plans are in place for Russia.

**Fig. 6:**
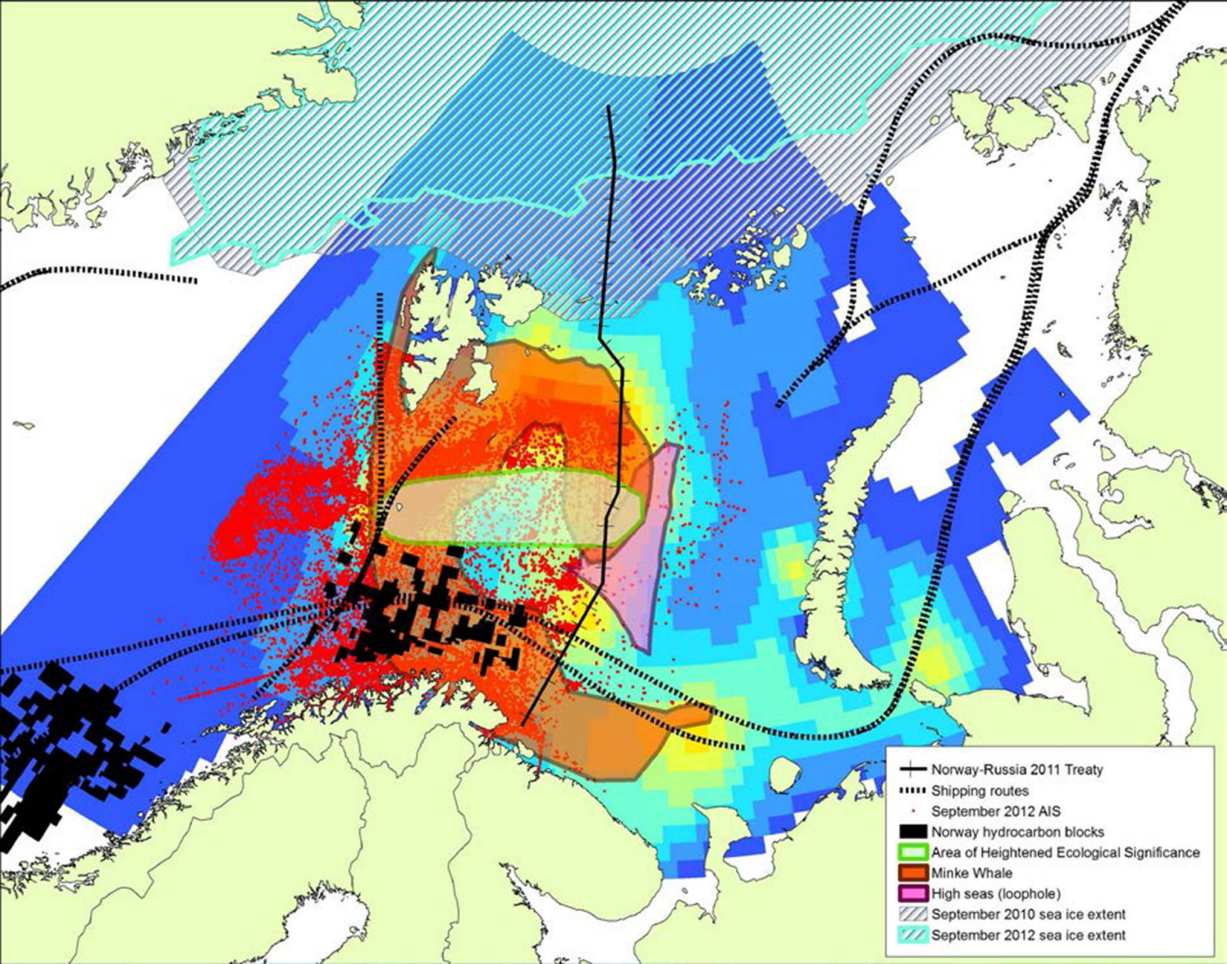
ArcGIS map showing different economic sectors in the Barents Sea. Coloured background grid shows predicted cod stocks for August 2057 (ACCESS report D3.11)—cold colours show low density, while warm colours show higher density. Red dots show vessel AIS data from September 2012 (ACCESS report D4.54), while dashed black lines show principal shipping routes. Maritime boundary between Norway and Russia is shown by the black ticked line, while the pink polygon shows the Loophole. Norwegian hydrocarbon exploration blocks are shown by black rectangles. Also shown are Minke Whale distribution, an ecologically significant area, and 2010 and 2012 summer sea-ice extents

We can use the MSP tool to analyse spatial and temporal data from all the economic sectors covered by ACCESS: fisheries, shipping and oil and gas (Fig. 6). Results from the ACCESS fisheries research work package show calculated cod (*Gadus morhua*) stock density for August 2057. The modelled results suggest that climate change does not lead to significant changes to cod stock from the present day, but do highlight that this area will continue to be a significant fisheries resource. Cod stocks are predicted to be high around the boundary between the Norwegian and Russian EEZs and the Loophole (area of high seas between Norway and Russia, and an area which has been subject to regional fishing disputes), and also further east in Russian waters offshore Novaya Zemlya, highlighting the need for transboundary management. The establishment of the Joint Norwegian-Russian Fisheries Commission in the 1970s has contributed towards sustainable management of the Barents Sea fisheries.

The hydrocarbon sector is well developed in both the Norwegian and Russian sectors of the Barents Sea. In 2011, the maritime boundary dispute between Russia and Norway in the Barents Sea was resolved, opening up new areas for hydrocarbon exploitation. The Barents Sea is undoubtedly a major hydrocarbon province and exploration is likely to extend further offshore in the future. Retreating sea ice may allow further northwards exploitation of hydrocarbons.

The Barents Sea is also an area of increasing shipping activity, which has recently seen large increases in the volume of petroleum products shipped along the Norwegian and Russian coasts. Shipping along the Northern Sea Route (including LNG) also passes through the Barents Sea. Reduction in summer ice extent is opening up the area to the north of Novaya Zemlya as a potential shipping route, which could significantly increase vessel traffic through the Barents Sea in southwest to northeast directions.

The Barents Sea is clearly an area facing increasing pressure from shipping, fisheries and oil and gas exploitation, and the need for spatial planning for sustainable development is clear. In addition to potential user–user conflicts, user–environment conflicts are highly probable too. For example, the distribution of Minke whales, as well as one of several identified areas of heightened ecological significance within the Barents Sea Large Marine Ecosystem, overlaps with areas of increased economic exploitation (Fig. 6). Minke whales are just one of many marine mammal species found in the Barents Sea. Increasing economic activity will lead to increased acoustic disturbance for marine mammals and possibly result in changes in their distribution. Pollution and vessel strikes are significant threats to marine wildlife and habitats too.

Figure 6 also highlights the need for transboundary MSP; geological (hydrocarbon) provinces, fish stocks and species distributions all cross borders. Equally, the effects of climate change will be seen on a regional scale. Truly effective MSP and EBM in the Arctic need to be considered at a pan-Arctic, multinational, scale (Ehler 2014).

## Conclusions

The ACCESS MSP tool has been developed to address a unique combination of climatic and geopolitical issues and is designed to provide a data integration system for the purposes of identifying or mitigating against possible future events or activities. The tool allows the integrated study of information from all sectors under review: hydrocarbon exploitation, shipping and fisheries, and the associated human activities related to and within these sectors. Using the concepts of Ecosystem-Based Management, recognition of sectoral uses and practical methodologies of data and relationships analyses with a powerful geographical information system, users of the MSP tool can visualise and assess in a qualitative way the factors relevant to sustainable development in the region, as they are affected by long-term climate change.

Marine spatial planning offers a transboundary, holistic approach to the governance of living and non-living resources. For this to succeed, there needs to be commitment at both national and regional levels. The involvement of stakeholders and users of the Arctic, including pan-national governance elements (such as the Arctic Council), is essential. Truly effective marine spatial planning needs to be considered at a multinational, Pan-Arctic scale.
